# Low-Dose Vitamin D Protects Hyperoxia-Induced Bronchopulmonary Dysplasia by Inhibiting Neutrophil Extracellular Traps

**DOI:** 10.3389/fped.2020.00335

**Published:** 2020-07-03

**Authors:** Cuie Chen, Huachun Weng, Xixi Zhang, Shi Wang, Chaosheng Lu, Hongxing Jin, Shujun Chen, Yuanyuan Liu, Anqun Sheng, Yuanyuan Sun

**Affiliations:** ^1^Department of Neonatology, Yiwu Maternity and Children Health Care Hospital, Jinhua, China; ^2^Chinese-American Research Institute for Pediatrics & Department of Pediatrics, The First Affiliated Hospital of Wenzhou Medical University, Wenzhou, China; ^3^Department of Pediatrics, Yuhuan People's Hospital, Taizhou, China; ^4^Department of Anesthesiology, Women's Hospital School of Medicine Zhejiang University, Hangzhou, China; ^5^Department of Pediatrics, The First Affiliated Hospital of Wenzhou Medical University, Wenzhou, China

**Keywords:** bronchopulmonary dysplasia, vitamin D, neutrophil extracellular traps, hyperoxia, histone

## Abstract

**Background and Objective:** As bronchopulmonary dysplasia (BPD) can lead to considerable mortality and morbidity, this disease is the focus of attention in neonatology. Vitamin D (VD), which has anti-inflammatory properties and promotes lung growth, may have a therapeutic effect on BPD. The overexpression of neutrophil extracellular traps (NETs) has been demonstrated to be involved in the pathogenesis of BPD in our previous study. This study aimed to elucidate the effect of VD on BPD and the role of NETs in this process.

**Methods:** Newborn rats were exposed to 90% oxygen continuously for 7 days to mimic BPD, and rats under hyperoxia were injected with 1,25(OH)2D3 at different doses (0.5 ng/g, 3 ng/g). Alveolarization, pulmonary vascular development, inflammatory cytokines and NETs were assessed.

**Results:** Hyperoxia increased mortality, decreased body weight, impaired alveolarization with a decrease in radial alveolar count (RAC) and an increase in mean linear intercept (MLI), and impaired vascular development with low vascular endothelial growth factor (VEGF) expression. Meanwhile, hyperoxia enhanced expression of the proinflammatory factors TNF-α, IL-1β, and IL-6, and elevated NETs in lung tissues and plasma. Low-dose VD (0.5 ng/g) administration increased the survival rate, attenuated developmental retardation, improved alveolarization, and pulmonary vascular development in hyperoxia-induced BPD, and reduced the expression of proinflammatory factors and NETs. However, high-dose VD (3 ng/g) treatment did not attenuate lung injury or NETs significantly, and even led to more severe developmental retardation and a higher mortality rate.

**Conclusions:** Low-dose VD increased the survival rate, attenuated developmental retardation, and improved alveolarization and pulmonary vascularization arrest in hyperoxia-induced BPD partially by inhibiting NETs.

## Introduction

Bronchopulmonary dysplasia (BPD) is a severe complication of extreme preterm birth that may affect pulmonary function, leading to pulmonary hypertension, mental retardation and growth retardation, or even cause considerable mortality, leading to a large health care burden (1, 2). The incidence of BPD increases annually due to improvement in the survival of extreme premature infants, but strategies to protect the lung are limited (1, 3). BPD is characterized by the arrest of alveolar and pulmonary vascular development. Chorioamnionitis, sepsis, hyperoxia, and invasive mechanical ventilation may contribute to this disease (1). Activation, infiltration and delayed clearance of polymorphonuclear neutrophils (PMNs), the most crucial innate immune cells, play important roles in the pathogenesis of BPD (4). Similarly, we found that neutrophil counts and the neutrophil-to-lymphocyte ratio (NLR) of patients with BPD were higher than those in individuals without BPD at birth and at 72 h. The neutrophil counts and NLR at 72 h of the group with severe BPD were higher than those in the group with mild BPD, and our previous study showed that an increase in the NLR at 72 h could be an early predictor of BPD, especially severe BPD (5).

Neutrophil extracellular traps (NETs) are extracellular, web-like structures composed of DNA, histones, myeloperoxidase (MPO), neutrophil elastase (NE), calprotectin, cathelicidins, defensins, and actin (6). NETs are stimulated by activated PMNs and can trap and kill invading pathogens under normal conditions. However, excess NETs formation and delayed NETs clearance lead to tissue and organ injury. NETs contribute to the pathogenesis of a variety of immune- and inflammation- related diseases, such as rheumatoid arthritis (7), acute lung injury (8), chronic obstructive pulmonary disease (9), and sepsis (10). In our previous study, we provided evidence that NETs were increased in hyperoxia-induced BPD rats, and anti-histone antibodies and heparin could attenuate lung injury by inhibiting NETs formation (11). Therefore, inhibition of NETs production may be a potential therapeutic approach in BPD.

Vitamin D (VD) is a secosteroid hormone, and its active form is 1,25(OH)2D3. Recently, VD was discovered to be an important modulatory molecule in immunity and inflammation in many organs (12–14). VD has also been shown to have a critical role in lung development, as it can increase surfactant synthesis and promote alveolar epithelial-mesenchymal interactions (15, 16). VD deficiency at an early stage of life is involved in BPD in very preterm infants. A low VD level at 24 h of life was shown to be a risk factor for the development of BPD (17), and a lower VD level was associated with the increased severity of BPD (18). Studies have demonstrated that VD could attenuate lung injury in an animal model of BPD through the suppression of interferon-gamma (IFN-γ) production (19) or downregulation of Toll-like receptor 4 (TLR4) (20); however, the mechanism of VD in BPD remains poorly understood and uncertain. Because VD can decrease NETs activity (21), we hypothesized that VD plays a protective role against BPD by regulating NETs.

In this study, we report alveolar and pulmonary vascular development and the inflammatory response after VD supplementation in hyperoxia-induced BPD and the effect of NETs on this process.

## Materials and Methods

### Animal Model and Study Design

Sprague Dawley rats (12 weeks old, 200–250 g, male:female=1:2 mating) were provided by the Shanghai Laboratory Animal Center. After natural pregnancy, each female rat was put into a separate cage. We used an established animal model of BPD that has been described previously (11). Briefly, after spontaneous delivery, the neonatal rats were exposed to hyperoxia (90% oxygen) with a 12-h light/dark cycle for 7 days to mimic BPD. The oxygen concentration was continuously monitored using an electronic recorder (EnviteC, Wismar, Germany). The CO_2_ in the environment was absorbed using soda lime. Dams were rotated between the hyperoxia and normoxia groups every 24 h. Cages were regularly opened for 30 min every day to replace the padding and provide clean drinking water and food, to which the rats had *ad libitum* access. The hyperoxia group was randomly divided into three subgroups, and the rats in these subgroups were treated with 1,25(OH)2D3 (Roche Pharma Ltd., Schweiz) at 0.5 ng/g or 3 ng/g i.p. once a day for 7 days or administered an equivalent volume of normal saline. The study was terminated on postnatal day 14 [P14]. The experimental protocol was approved by the Experimental Animal Ethics Committee of Wenzhou Medical University (wydw2019-0957).

### Lung and Blood Processing

On P7 and P14, 6 newborn rats from each group were anesthetized by i.p. injection of 5% chloral hydrate (10 g in 0.05 ml), and blood samples then were aseptically collected by arteria carotis communis puncture and placed into tubes with sodium citrate (1:10) on ice. The samples were centrifuged at 3,000 rpm for 30 min at 4°C, and the plasma was then stored at −80°C. The whole lungs were aseptically collected by an open-chest procedure. Samples of the right lung were kept at −80°C. The left lung was perfused with 4% polyformaldehyde at a pressure of 20 cm H_2_O and then fixed in a 4% paraformaldehyde solution at 4°C.

### Lung Morphometry

Lungs were sectioned at a thickness of 4 μm and stained with hematoxylin and eosin (H&E) to reveal the lung morphometry. Three random non-overlapping fields in one distal lung section per rat were utilized for morphometric examinations. The radial alveolar counts (RAC) were measured according to Cooney and Thurlbeck, who proposed drawing a perpendicular line from the center of the most peripheral bronchiole to the pleura or the nearest interlobular septum and counting the number of alveoli transected by this line (22). The mean linear intercept (MLI) was also determined to evaluate alveolar size (14, 23).

### Measurement of Pulmonary Vascular Development by Immunohistochemistry

Vascular endothelial growth factor (VEGF) expression in the lung tissue was detected by immunohistochemistry. First, the sections were deparaffinized in xylene, rehydrated, and rinsed in deionized water. Then, antigen retrieval was performed by microwave treatment in EDTA buffer (pH 9.0) for 8 min. The slides were cooled and held at room temperature for 15 min. Sections were washed with phosphate-buffered saline (PBS) (pH 7.4), and endogenous peroxidase activity was blocked in 3% H_2_O_2_ for 25 min. Next, the sections were incubated with anti-VEGF antibody (Ab-1, goat polyclonal antibody, Abcam, USA) diluted 1:100 in antibody diluent buffer overnight at 4°C. After washing with PBS, sections were incubated with biotinylated HRP-labeled secondary antibodies (GB23301, Servicebio, China). The sections were visualized using a 3,3′-diaminobenzidine tetrahydrochloride chromogen kit (K5007, DAKO, Danish), and the cell nuclei were stained again with hematoxylin. To remove water, an increasing alcohol series and xylene were used. Finally, the sections were covered with a slide with neutral gum. From each of the sections, four different fields were selected under the microscope (200×), and the average optical density (AOD) of stained VEGF was determined by Image-Pro Plus 6.0 image analysis software.

### Evaluation of Cytokine Levels

Lung samples were kept at −80°C until use. The samples were homogenized on ice and centrifuged at 12,000 rpm for 30 min, and the supernatants were collected for analyses. The tissue levels of tumor necrosis factor-alpha (TNF-α), interleukin-1Beta (IL-1β) and interleukin-6 (IL-6) were measured with an ELISA kit (Ray Biotech, Inc., Guangzhou) following the manufacturer's instructions.

### NETs Detection by Immunofluorescence Staining

The sections were fixed, stained and imaged to detect NETs. The sections were incubated with the specific primary antibodies anti-citrullinated histone 3 (Cit-H3) (1:100; SC51716, Santa Cruz) and anti-MPO (1:500; GB11224, Google biological) overnight at 4°C. Then, the sections were incubated with Alexa Fluor 488-conjugated goat anti-rabbit (1:400; GB21303, Google biological) and Alexa Fluor Cy3-conjugated goat anti-mouse (1:300; GB21301, Google biological) secondary antibodies for 1 h. 4′,6-Diamidino-2-phenylindole (DAPI, G1012, Google biological) was used to detect DNA. All the slides were visualized using an Olympus FluoView 500 confocal scanning laser microscope.

### Quantification of Plasma NETs

Plasma NETs were detected by the NET-specific markers cell-free DNA and Cit-H3. The plasma cell-free DNA was quantified with a Quant-iT PicoGreen dsDNA Assay Kit (Invitrogen, Canada) following the manufacturer's protocol. A 50-μl dilution of a standard solution was added to 96-well plates, and then 100 μl of PicoGreen fluorescent dye was added before incubation at room temperature away from light for 5 min. Fluorescence intensity was detected by a fluorescence plate reader, and the cell-free DNA/NETs were quantified. Cit-H3 was measured with a Cell Death Detection ELISA PLUS kit (Roche, Switzerland). An appropriately diluted sample (1:500) was added to the plates and incubated at room temperature for 2 h. Then, the substrate was added and incubated for 30 min, and a chromogenic agent was added and incubated for 15 min. The absorbance at 490 nm and 405 nm was captured, and the difference in OD at 405 nm and 490 nm was calculated as the relative quantitative value of Cit-H3.

### Statistics Analysis

The results are presented as the mean ± standard deviation. The data were analyzed by SPSS 25.0 (SPSS, Inc., Chicago, IL, USA). Two-group comparisons were carried out using Student's *t*-test. Comparisons of more than two groups were carried out using one-way ANOVA. A *P* < 0.05 indicated statistical significance.

## Results

### Low-Dose Vitamin D Promoted the Survival Rate and Attenuated Developmental Retardation in Hyperoxia-Induced BPD

**Figure 1:**
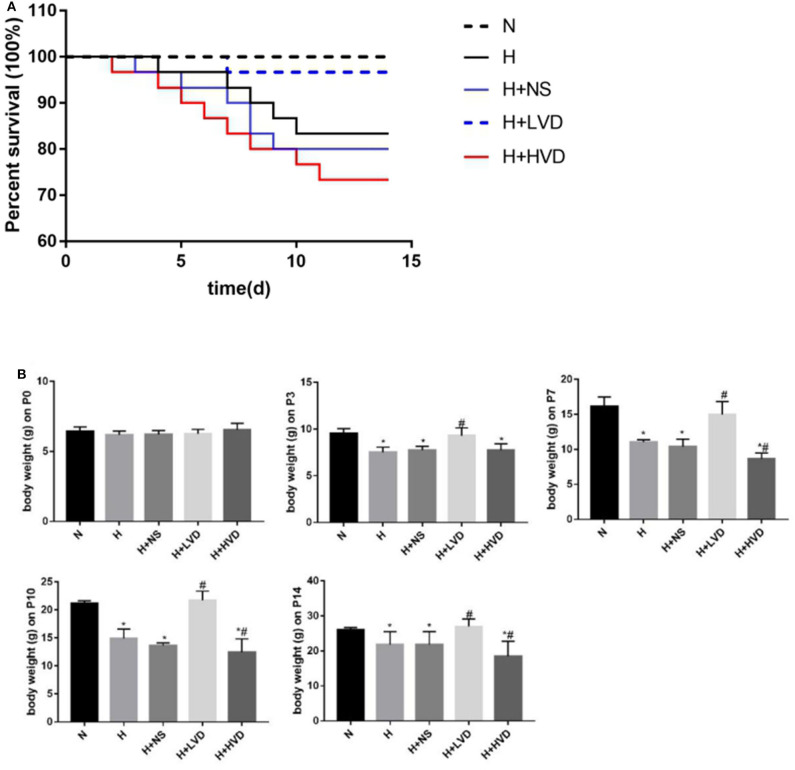
LVD (0.5 ng/g) treatment increased survival and attenuated developmental retardation in hyperoxia. **(A)** Kaplan-Meier survival analysis (*P* < 0.05). **(B)** The body weight in different groups. *P < 0.05, compared to normoxia group (N); ^#^*P* < 0.05, compared to hyperoxia group (H).

*In vivo* treatment of rats with low-dose VD (LVD) (0.5 ng/g) significantly reduced the mortality caused by hyperoxia, which was not different from that in the normoxia group. Only 83.3% of the rats survived hyperoxia, while LVD increased the rate to 96.7%, but the survival rate of rats treated with high-dose VD (HVD) (3 ng/g) was 73.3% (Figure 1A). The rats in the hyperoxia group presented with developmental retardation, and a decreased body weight compared to that of the control groups was observed from P3. Treatment of the rats with LVD significantly attenuated this retardation, but treatment of the rats with HVD led to more severe developmental retardation (Figure 1B).

### Low-Dose Vitamin D Improved Alveolar Development in Hyperoxia-Induced BPD

**Figure 2:**
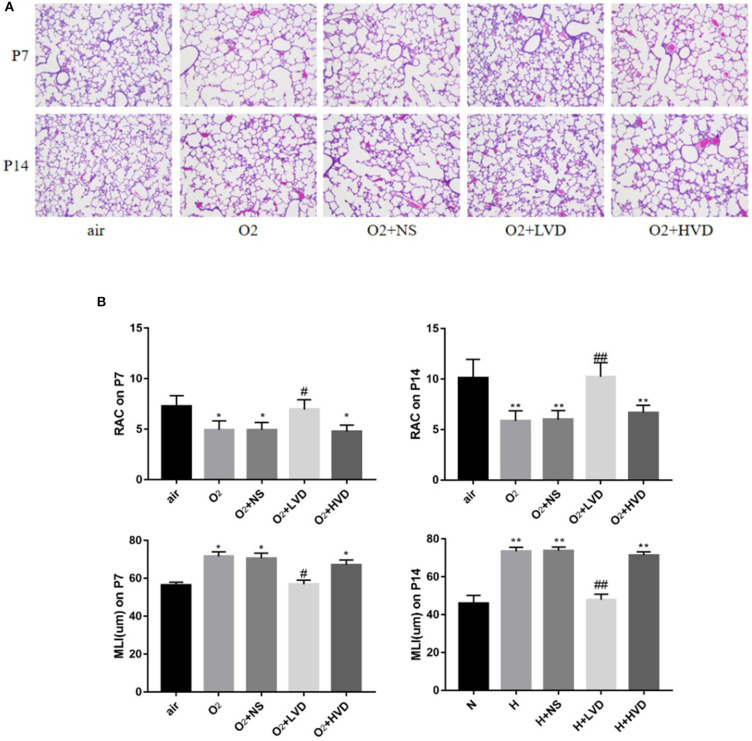
LVD (0.5 ng/g) treatment improved alveolarization in hyperoxia-induced BPD. **(A)** The H&E assessment of lung tissues. Magnification, ×200. **(B)** The RAC and MLI value. *P < 0.05, **P < 0.01, compared to normoxia group (N); ^#^*P* < 0.05,^*##*^*P* < 0.01, compared to hyperoxia group (H). *n* = 6.

The lung structures of rats in the hyperoxia group had characteristics typical of alveolar simplification, as indicated by enlarged alveoli with decreased terminal alveoli and secondary septa, as well as irregular alveolar shape and thickening of the alveolar wall (Figure 2A). In the rescue experiment, the rats treated with LVD (0.5 ng/g) exhibited improved alveolarization after hyperoxia exposure, but the rats treated with high-dose HVD (3 ng/g) did not exhibit attenuated lung injury (Figure 2A). These effects were achieved by an increase in RAC and a decrease in MLI (Figure 2B).

### Low-Dose Vitamin D Improved Pulmonary Vascular Development in Hyperoxia-Induced BPD

**Figure 3:**
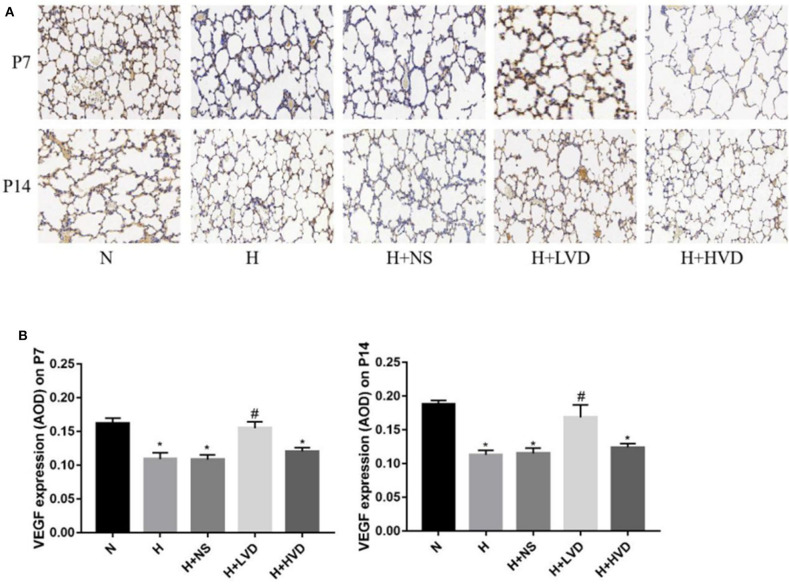
LVD (0.5 ng/g) treatment improved lung angiogenesis. **(A)** The immunohistochemical assessment for VEGF protein. Magnification, ×200. **(B)** The AOD value of VEGF. *P < 0.05, compared to normoxia group (N); ^#^*P* < 0.05, compared to hyperoxia group (H) *n* = 6.

Compared to rats in the normoxia group, rats in the hyperoxia group exhibited decreased lung VEGF expression, as shown by immunohistochemistry (Figure 3A). The rats treated with LVD exhibited significantly improved pulmonary vascular development due to significantly increased protein expression of VEGF (Figure 3B). In contrast, the rats treated with HVD did not exhibit increased expression of VEGF under hyperoxia.

### Low-Dose Vitamin D Reduced Inflammation in Hyperoxia-Induced BPD

**Figure 4:**
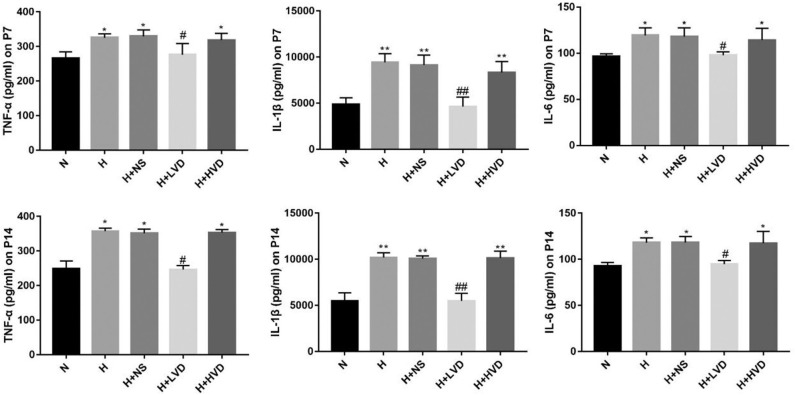
LVD (0.5 ng/g) treatment reduced proinflammation factors in hyperoxia-induced BPD rats on P7 and P14. *P < 0.05, **P < 0.01, compared to normoxia group (N); ^#^*P* < 0.05, ^*##*^*P* < 0.01, compared to hyperoxia group (H) *n* = 6.

Exposure of newborn rats from birth to P7 to hyperoxia (90%) resulted in increased expression of the inflammatory cytokines TNF-α, IL-1β, and IL-6 in the lung tissues, and LVD treatment significantly decreased the levels of these inflammatory cytokines, but the rats administered HVD i.p. did not exhibit an inhibited inflammation response (Figure 4).

### Low-Dose Vitamin D Inhibited NETs Formation in Hyperoxia-Induced BPD

**Figure 5:**
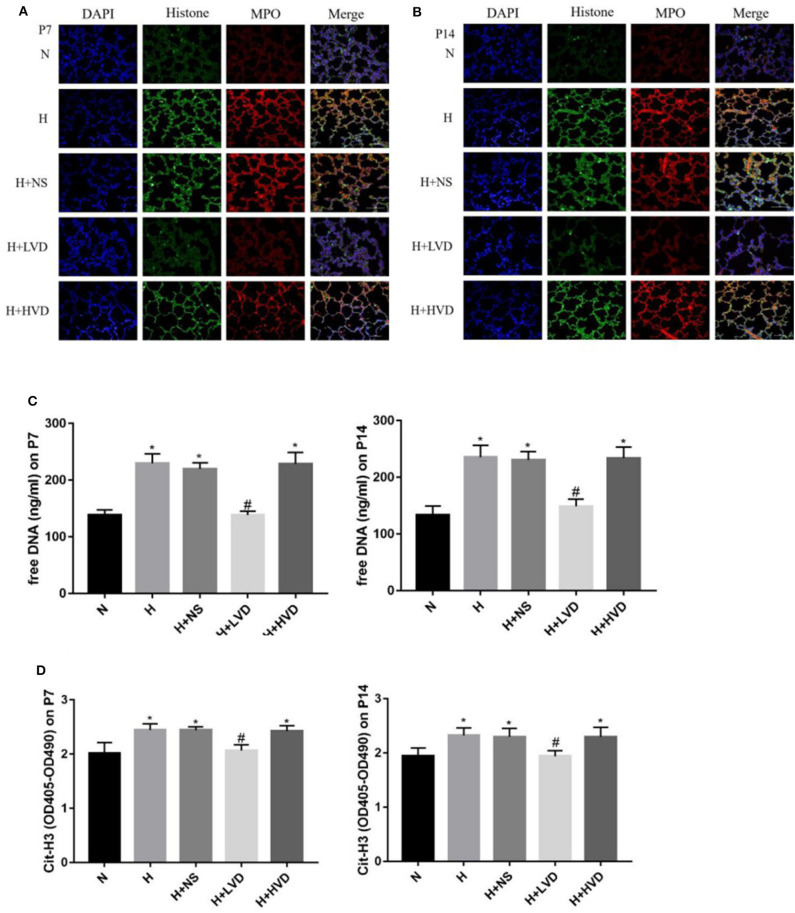
LVD (0.5 ng/g) treatment reduced NETs formation in hyperoxia-induced BPD rats **(A,B)**. The NETs formation identified by immunofluorescence staining. Magnification, ×200. **(C)** The expressions of free DNA in plasma. **(D)** The expressions of circulating histones. *P < 0.05, compared to normoxia group (N); ^#^*P* < 0.05, compared to hyperoxia group (H) *n* = 6.

To investigate the NETs formation and changes, Cit-H3, MPO, and DNA, which are known markers of NETs formation, in lung tissues were identified by immunofluorescence staining. Marked staining for Cit-H3, MPO, and DNA was observed in rats under hyperoxia but not rats under normoxia (Figures 5A,B). The treatment of rats with LVD inhibited NETs formation, as manifested by decreased staining. Plasma cell-free DNA and Cit-H3 were markedly elevated in the hyperoxia group compared with the normoxia group (Figures 5C,D). LVD treatment significantly decreased the level of cell-free DNA and Cit-H3 expression in rats exposed to hyperoxia, but HVD treatment did not obviously reduce NETs formation.

## Discussion

Lung development in extremely preterm infants occurs in the transition from the canalicular to saccular stages, which results in a gas-exchange barrier and leaves extremely preterm infants at high risk of neonatal respiratory distress syndrome (NRDS). To improve the survival rates of extremely preterm infants, it is necessary to use supplemental oxygen or mechanical ventilation for respiratory failure. However, these measures induce further lung injury and interrupt pulmonary alveolar and vascular development, eventually contributing to BPD. The incidence of BPD, as demonstrated by major cohort studies, is 11–50% and increases with decreased gestational age and birth weight (1). Due to the increase of *in vitro* fertilization (IVF), multiple births and preterm infants in recent years, BPD is associated with high morbidity and mortality due to its limited effective therapeutics and thus represents a major public health concern, drawing attention worldwide.

The pathogenesis of BPD is multifactorial; however, inflammation is generally believed to be the primary mediator of lung injury in preterm infants (24). Inflammatory signaling pathways, such as the TLR4 and NF-κB pathways, were indicated to be associated with BPD (21, 25). The levels of proinflammatory cytokines, such as TNF-α, IL-1β, IL-6, and IL-8, are increased, and the levels of anti-inflammatory cytokines, such as IL-4, IL-10, IL-12, IL-13, and IL-18, are decreased in BPD (26, 27). Pulmonary inflammation in BPD is characterized by the presence of inflammatory cells, PMNs, monocytes, proinflammatory cytokines, and soluble adhesion molecules (25). Neutrophil influx into the airways and pulmonary edema formation occur after exposure to hyperoxia or mechanical ventilation, increasing the risk of developing BPD. PMNs are activated and adhere to epithelial cells of alveoli and the endothelium of the pulmonary vasculature during the inflammatory process, initiating a series of pulmonary injuries. Activated PMN exosomes play an important role in chronic inflammatory diseases in the lung and have been demonstrated as the pathogenic entities causing BPD (4). Furthermore, neutrophils undergo apoptosis at a slower pace and cannot be cleared in a timely manner in preterm neonates, prolonging neutrophil survival and aggravating lung inflammation and BPD (26).

Activated PMNs expel decondensed chromatin with embedded inflammatory proteins, known as NETs, which have been shown to damage tissues and organs in infection and sterile inflammatory disease upon dysregulation (28). NETs and histones can directly induce the release of several proinflammatory cytokines, including TNF-α, IL-1β, and IL-6 (29). NETs release occurs primarily through a cell death process termed NETosis (30). Furthermore, excessive NETosis can compromise cell membrane integrity, disrupt cell junctions, and kill epithelial and endothelial cells, effects that have been described in pulmonary diseases such as pulmonary infection, acute lung injury and COPD (28, 31, 32). Studies demonstrated that the disruption of NETs by DNase I administration resulted in decreased disease severity in mouse models of transplantation-related lung injury (33), and anti-histone antibodies could improve lung injury and survival in a mouse model of a lipopolysaccharide-induced sepsis-like syndrome (34). We previously investigated NETs in an animal model of BPD and showed that inhibition of NETs with anti-histone antibodies or heparin reduced lung injury (11), which also implicated antagonizing the activation of NETs as a potential therapeutic approach in BPD.

In recent decades, VD has been shown to be involved in the modulation of immunity, inflammation, infection, fibrosis and cancer, in addition to its traditional role in regulating calcium and phosphorus homeostasis (12, 13, 35). VD can suppress neutrophil activation, induce antimicrobial peptides, and reduce the production of inflammatory mediators and reactive oxygen intermediates in PMNs (36, 37). VD also plays a crucial role in cellular growth and differentiation, including the regulation of lung maturation (15). Moreover, the impact of VD on early lung development and BPD has become an emerging field of research in recent years (18, 20). Yao et al. (20) suggested that VD attenuated hyperoxia-induced lung injury by protecting the integrity of the lung structure, decreasing extracellular matrix deposition, inhibiting inflammation, and antagonizing the activation of TLR4. Kose et al. (14) demonstrated that treatment with VD protected against hyperoxia-induced lung injury by decreasing MLI and apoptosis and increasing the proliferating cell nuclear antigen (PCNA) index. Liu et al. (19) suggested that supplementation with VD could enhance alveolar development in an LPS-induced BPD rat model through the suppression of IFN-γ production. However, whether other regulatory mechanisms are involved in VD as a treatment in hyperoxia-induced lung injury remains unknown. Moreover, VD was proven to have the capacity to inhibit the formation of phagocytic oxidase (Phox), which is a key enzyme in the formation of NETs (38). Handono et al. (21) demonstrated that VD treatment could decrease NETs activity in patients with systemic lupus erythematosus (SLE) to prevent endothelial damage.

In our study, we found that newborn rats exposed to hyperoxia had increased mortality, decreased body weight, and restricted lung development, resulting in fewer enlarged alveolar air spaces, the loss of lung capillaries, and increased inflammatory factors. We began our investigation with an evaluation of lung tissue and plasma samples for the NETs markers MPO, Cit-H3, and cell-free DNA and found that their levels were significantly elevated in hyperoxia-induced BPD rats. At the same time, treatment with LVD increased the survival rate, attenuated developmental retardation, improved alveolar and lung vascular growth by increasing the RAC, decreasing MLI, upregulating VEGF expression, reducing the production of inflammatory cytokines, and inhibiting the formation of NETs. However, treatment with HVD did not significantly attenuate lung injury or NETs and even led to more severe developmental retardation and a higher mortality rate. Interestingly, Kose et al. (14) found that 1,25(OH)2D3 has more therapeutic potential at higher dosages (3 ng/g/day and 5 ng/g/day) than at a dosage of 1 ng/g/day. In contrast, we observed that LVD, but not HVD, had therapeutic effects. Furthermore, we demonstrated that LVD protected against hyperoxia-induced lung injury by inhibiting the formation of NETs, which has not been reported in previous studies. We believe that LVD inhibit NETs and further inhibit the release of proinflammatory factors, thus improving lung injury caused by hyperoxia, which is part of the mechanism of the treatment of BPD with LVD. We also observed weight loss as a side effect of HVD. Abbas (39) suggested that VD regulates adipogenesis at various stages of the differentiation process and certain molecular factors. Therefore, we speculate that VD affects the key genes in adipose metabolism and lung development, which will be confirmed in our future studies.

This study has several limitations. BPD is a complex, multifactorial disease that may only be partially explained by the effects of hyperoxia exposure, and antenatal infection also needs to be considered in a future study. In addition, further work is needed to determine the most effective dosage of VD in an animal model or in a clinical setting. Finally, future studies are needed to address the effects of VD exposure on metabolic functions, which may play an important role during lung development.

In conclusion, administration of LVD (0.5 ng/g) increased the survival rate, attenuated developmental retardation, improved alveolarization and pulmonary vascular development, and alleviated inflammation in hyperoxia-induced BPD partially by inhibiting NETs.

### What Is Known

Bronchopulmonary dysplasia (BPD) is a severe complication in extreme preterm infants. Vitamin D (VD) has anti-inflammatory properties, promotes lung growth, and may have a therapeutic effect on BPD. In our previous study, neutrophil extracellular traps (NETs) overexpression was demonstrated to be involved in the pathogenesis of BPD.

### What Is New

In the present study, we found that low-dose VD increased the survival rate, attenuated developmental retardation, and improved alveolarization and pulmonary vascularization arrest in hyperoxia-induced BPD partially by inhibiting NETs.

## Data Availability Statement

The raw data supporting the conclusions of this article will be made available by the authors, without undue reservation, to any qualified researcher.

## Ethics Statement

The experimental protocol was approved by the ethics committee of Wenzhou Medical University (wydw 2019-055).

## Author Contributions

CC provided the idea, managed the experiments, analyzed the results, involved in manuscript preparation, and provided the fund. HW designed experiment, managed the experiments, analyzed the results and involved in manuscript preparation. XZ and SW was involved in animal experiments and samples test. CL and HJ was involved in data collection and analysis. SC analyzed the results, and was involved in manuscript preparation. YL was involved in animal experiments. AS was involved in samples test. YS provided the idea of this experiments and managed it. All authors contributed to the article and approved the submitted version.

## Conflict of Interest

The authors declare that the research was conducted in the absence of any commercial or financial relationships that could be construed as a potential conflict of interest.

## Abbreviations

BPD: bronchopulmonary dysplasia
VD, Vitamin D/1: 25(OH)2D3
NETs: neutrophil extracellular traps
RAC: radial alveolar count
MLI: mean linear intercept
VEGF: vascular endothelial growth factor
PMNs: polymorphonuclear neutrophils
NLR: neutrophil-to-lymphocyte ratio
VLBWI: very low birth weight infants
MPO: myeloperoxidase
NE: neutrophil elastase
H&E: hematoxylin and eosin
PBS: phosphate-buffered saline
AOD: average optical density
IFN-γ: interferon-gamma
TNF-α: tumor necrosis factor-alpha
IL-1β: interleukin-1Beta
IL-6: interleukin-6
Cit-H3: anti-citrullinated-histone 3
DAPI 4′: 6-diamidino-2- phenylindole
LVD: low-dose VD
HVD: high-dose VD
NRDS: neonatal respiratory distress syndrome
COPD: chronic obstructive pulmonary disease
Phox: phagocytic oxidase
PCNA: proliferating cell nuclear antigen
SLE: systemic lupus erythematosus.
